# Color attributes, betacyanin, and carotenoid profiles, bioactive components, and radical quenching capacity in selected *Amaranthus gangeticus* leafy vegetables

**DOI:** 10.1038/s41598-021-91157-8

**Published:** 2021-06-02

**Authors:** Umakanta Sarker, Shinya Oba

**Affiliations:** 1 Department of Genetics and Plant Breeding, Faculty of Agriculture, Bangabandhu Sheikh Mujibur Rahman Agricultural University, Gazipur, 1706 Bangladesh; 2 Laboratory of Field Science, Faculty of Applied Biological Sciences, Gifu University, Yanagido 1-1, Gifu, Japan

**Keywords:** Biochemistry, Natural variation in plants

## Abstract

Four selected *A. gangeticus* accessions were evaluated in terms of color attributes, phytopigments, including betaxanthin, betacyanin, and carotenoid profiles, proximate, minerals, and antioxidant capacity (AC). Color attributes, phytopigments, proximate, minerals, and AC of *A. gangeticus* significantly varied across the accessions. For the first time, we identified four betacyanin compounds, such as amaranthine, iso-amaranthine, betanin, iso-betanin. We also identified five carotenoid compounds zeaxanthin neoxanthin, violaxanthin, lutein, and pro-vitamin A in *A. gangeticus* accessions. *A. gangeticus* contained adequate carbohydrates, protein, moisture, and dietary fiber. We found adequate iron, manganese, copper, zinc, sodium, molybdenum, boron, potassium, calcium, magnesium, phosphorus, sulfur in *A. gangeticus* accessions. The accessions LS7 and LS9 had considerable color attributes, betacyanin, and carotenoid compounds, proximate, nutraceuticals, betalain, betaxanthin, and AC that could be used as preferable potent antioxidant varieties for consumption as sources of phytopigments, nutraceuticals, and antioxidants. The correlation study revealed that antioxidant constituents of *A. gangeticus* accession were strongly associated with AC. The identified components of betacyanin and carotenoid in *A. gangeticus* demands detail pharmacological study. The baseline data on color attributes, betacyanin, and carotenoid profiles, betaxanthins, betalains, and AC obtained in this present study could contribute to the scientific evaluation of pharmacologically active principles in *A. gangeticus*.

## Introduction

The young edible fleshy stems and baby leaves of amaranths are inexpensive and excellent sources of protein containing essential amino acids lysine and methionine, dietary fiber, carotenoids, vitamin C, minerals^1–6^. It has considerable pigments including carotenoids, betacyanins, betaxanthins, anthocyanins, chlorophylls, and betalains with high antioxidant capacity (AC)^7–10^, antioxidant compounds including phenolic acids, vitamin C, and flavonoids with high AC^11–14^. These antioxidant compounds quench reactive oxygen species (ROS) in the human body and play a significant contribution to the industry of food by protecting several diseases, including cataracts, cancer, cardiovascular diseases, emphysema, atherosclerosis, retinopathy, arthritis, and neurodegenerative diseases^15–18^.

Taste, color, and flavor of foods are the essential factor that primarily determines the acceptability of foods. Hence, currently, colored-food products gain the common interest of the people of the globe. Consumers are interested in colored-food products due to the aesthetic, nutritional, and safety aspects of foods, which have increased the demand for natural pigments such as betacyanins, including amaranthine, iso-amaranthine, betanin, iso-betanin, anthocyanins, carotenoids, and chlorophylls. The leafy vegetable amaranth is considered a unique source of betacyanins that have significant free radical-quenching capacity^19^. Betacyanins have higher pH stability than anthocyanins that can be used as a colorant in low-acid foods^20^. Amaranths have amaranthine (a primary pigment of betacyanins), including high AC. It can substitute the betanins of red beets as natural antioxidants and food colorants^21^. Amaranths are widely adapted vegetables in drought^22–25^ and salinity^26–29^. Red and maroon color amaranth have more pigments, including betacyanins and carotenoids than green color amaranth. Amaranths leaves prohibit the proliferation of liver (HepG2), breast (MCF-7), and colon (Caco-2) cancer cell lines, including anticancer potential^30^.

Children and adults in Bangladesh, including many developing countries, face severe threats of vitamin A deficiency and age-related macular degeneration. Age-related macular degeneration is increasing at an alarming rate in many developing countries. Vitamin A deficiency and age-related macular degeneration are mainly due to inadequate consumption of pro-vitamin A and macular pigments in the daily diet. Hence, we can eradicate vitamin A deficiency and age-related macular degeneration by regular consumption of vitamin A enriches vegetables. *A. gangeticus* contains high β-carotene that could be used as an inexpensive natural source of pro-vitamin A. Different climatic conditions in the globe remarkably influence the carotenoid profiles of crops. Even different eco-geographical regions of the same country varied the carotenoid profiles of the same species. Currently, we are evaluating the chances of utilizing *A. gangeticus* pigments profile containing considerable natural macular pigments with high antioxidant potentials of interest in the industry of foods^17,21^. We previously screened few *A. gangeticus* accessions based on yields and AC and selected the best four high-yielding and antioxidant potential accessions LS3, LS5, LS7, and LS9. For the first time, the color attributes, betacyanins, and carotenoid profiles, and antioxidants potentials in *A. gangeticus* were studied in detail through spectrophotometry, HPLC, and LC–MS. Therefore, we ultimately evaluate the possibility of selection of appropriate accessions for extracting colorful juice for drink purposes with high color attributes, betacyanins and carotenoids, profile, and antioxidants potentials.

## Results and discussion

The evaluated traits demonstrated a wide range of variations across the *A. gangeticus* accessions. A wide range of variability was also reported in vegetable amaranth^2^, rice^31–45^, maize^46–48^, and coconuts^49–50^.

### Color attributes

**Figure 1:**
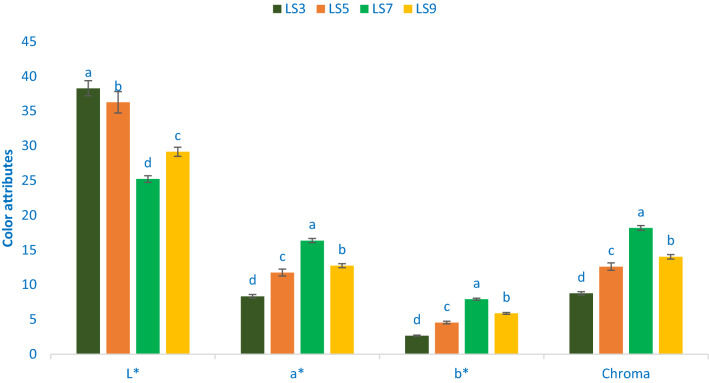
Color attributes in four selected *A. gangeticus* leafy vegetables, L*, Lightness; a*, Redness/greenness; b*, Yellowness/blueness, different letters in the bar are differed significantly by Duncan Multiple Range Test ((P < 0.01), (n = 3).

Figure 1 shows the color attributes of leaf four of selected *A. gangeticus* leaves. The variations in terms of chroma, lightness (L*), redness/greenness (a*), and yellowness/blueness (b*) in four studied accessions were significant and prominent. The range of chroma, lightness (L*), a*, and b* were 8.76 to 18.18, 25.22 to 38.28, 8.34 to 16.36, and 2.67 to 7.35, respectively. The accession LS3 exhibited the highest value of lightness (38.28), while the accession LS7 had the lowest lightness (25.22), followed by LS9 (29.15). Likewise, b* (7.92), a* (16.36), and chroma (18.18) values were the highest the accession LS7 followed by LS9, while the accession LS3 showed the lowest b* (2.67), a* (8.32), and chroma (8.76) values. Colonna et al.^51^ and our previous study in red and green color amaranth^52^ were corroborated with our present study of *A. gangeticus*. The essential attributes, choice, preference, and acceptability of consumers are broadly and significantly by color attributes, tests, and nutritional quality of vegetables. Across them, color is the most vital indicator for evaluating antioxidant potentials of leafy vegetables^51^. The accessions LS7 and LS9 with deep red color exhibited high values of yellowness and redness that signifies the presence of adequate pigments (betaxanthin, betalain, carotenoids, betacyanin, and anthocyanins). Conversely, the accession LS3 had low pigments (betaxanthin, betalain, carotenoids, betacyanin, and anthocyanins) as this accession exhibited low yellowness and low redness. The bright red-violet color accession containing adequate pigments, including betacyanin, has better stability at lower temperatures (< 14 °C) at pH 5–7^53^. We can use these accessions to extract colorant natural preservatives from food products and colorful drinks.

### Betacyanin components

**Figure 2:**
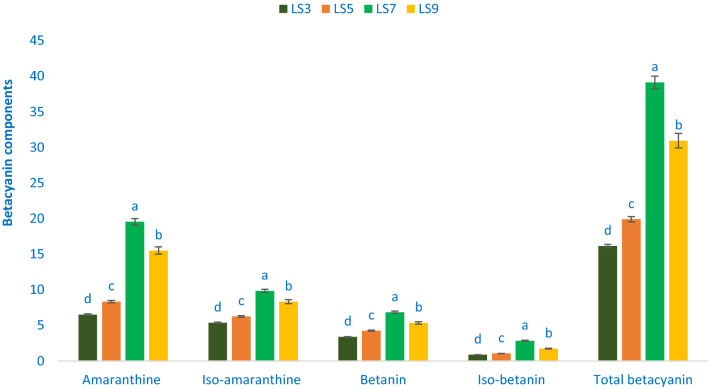
Betacyanin profiles in four selected *A. gangeticus* leafy vegetables, Amaranthine (mg 100 g^−1^), Iso-amaranthine (mg 100 g^−1^), Betanin (mg 100 g^−1^), Iso-betanin (mg 100 g^−1^), Total betacyanin (mg 100 g^−1^), different letters in the bar are differed significantly by Duncan Multiple Range Test ((P < 0.01), (n = 3).

The red-violet and maroon color *A. gangeticus* accessions contain high betacyanin pigments. Table 1 shows the data on the λmax, molecular ion, main fragment ions in MS^2^, retention time, and identified betacyanin compounds. The values of betacyanin compounds from four accessions (LS3, LS5, LS7, and LS9) separated through LC were compared with standard betanin (we used betanin of red beet as retention time standards) masses with respective peaks of the compounds. Figure 2 shows the betacyanin compounds and their qualified proportions in the leaf extracts. The HPLC detected betacyanin showed 2 principal peaks at 538 nm (peaks 1 and 2) (Table 1). Peak 1 and peak 2 had a higher degree of glycosylation as these peaks eluted earlier than standards betanin and iso-betanin (peaks 3 and 4)^54^. *Amaranthus* had abundant pigments (betacyanin compounds) such as iso-amaranthine and amaranthine^55^. Cai et al.^56^ also noticed that iso-amaranthine (its C15 epimer) and amaranthine were found in 37 *Amaranthus* species in 8 genera (91.5% of the 40 accessions). Betacyanin compounds such as betanin and amaranthine have the same aglycone unit betanidin. Amaranthine has glucuronosylglucoside, while betanin has glucoside. The identified molecule aglycone ion for peaks 3 and 4 had major fragment ion at *m/z* 389 [M-glucose+H]^+^, both peaked at *m/z* 551 [M+H]^+^ corresponding to the mass of betanidin glucoside. In *A. gangeticus* extract, the molecule corresponding to betanidin glucuronosylglucoside for peaks 1 and 2 was the same at *m/z* 727 [M+H]^+^. The major fragment ions for peaks 1 and 2 were also the same at *m/z* 551 [M-glucuronic acid+H]^+^ and with the same aglycone ion at *m/z* 389 [M-glucuronosylglucose+H]^+^ (Table 1). Based on the UV–Vis and MS data showed in Table 1, and those reported by others peaks 1 and 2 were identified tentatively as amaranthine and iso-amaranthine (C15 epimer), respectively^53–54^.

**Table 1:** Retention time (Rt), wavelengths of maximum absorption in the visible region (λ_max_), mass spectral data and tentative identification of betacyanin component in four selected *A. gangeticus* leafy vegetables.

Peak no	Rt (min)	λ_max_ (nm)	Molecular ion [M−H]^−^ (m/z)	MS^2^ (m/z)	Identity of tentative betacyanin component
1	1.56	538	727	551, 389	Amaranthine (betanidin 5-*O*-β-glucuronosylglucoside)
2	1.82	538	727	551, 389	Iso-amaranthine (isobetanidin 5-*O*-β-glucuronosylglucoside)
3	2.26	538	551	389	Betanin (betanidin 5-*O*-β-glucoside)
4	2.72	538	551	389	Iso-betanin (isobetanidin 5-*O*-β-glucoside)

Within betacyanin compounds, amaranthine was identified as the most prominent betacyanin, followed by iso-amaranthine in *A. gangeticus* accession (Fig. 2). Betanin, amaranthine, iso-amaranthine, and iso-betanin obtained from *A. gangeticus* accessions LS7 and LS9 were higher than the results in the stem of *A. spinosus* of Stinzing et al.^54^. The range of betanin, amaranthine, iso-amaranthine, total betacyanin, and iso-betanin were 0.87 to 2.84, 6.52 to 19.57, 5.38 to 9.85, 16.14 to 39.11, and 3.37 to 6.84, mg 100 g^−1^, respectively (Fig. 2). The highest betanin (6.84 mg 100 g^−1^), amaranthine (19.57 mg 100 g^−1^), iso-amaranthine (9.85 mg 100 g^−1^), total betacyanin (39.11 mg 100 g^−1^), and iso-betanin (2.85 mg 100 g^−1^) were obtained from the accession LS7, followed by the accession LS9. On the other hand, lowest iso-amaranthine (5.38 mg 100 g^−1^), amaranthine (6.52 mg 100 g^−1^), betanin (3.37 mg 100 g^−1^), total betacyanin (16.14 mg 100 g^−1^), and iso-betanin (0.87 mg 100 g^−1^) were noticed in the accession LS3.

### Carotenoid profiles

**Figure 3:**
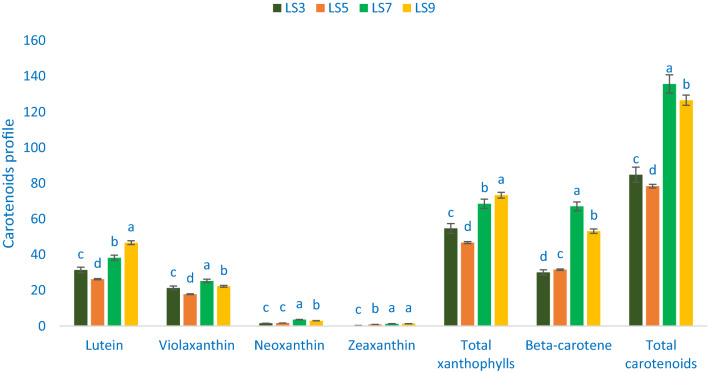
Carotenoid profiles (mg 100 g^−1^ FW) in four selected *A. gangeticus* leafy vegetables, different letters in the bar are differed significantly by Duncan Multiple Range Test ((P < 0.01), (n = 3).

**Figure 4:**
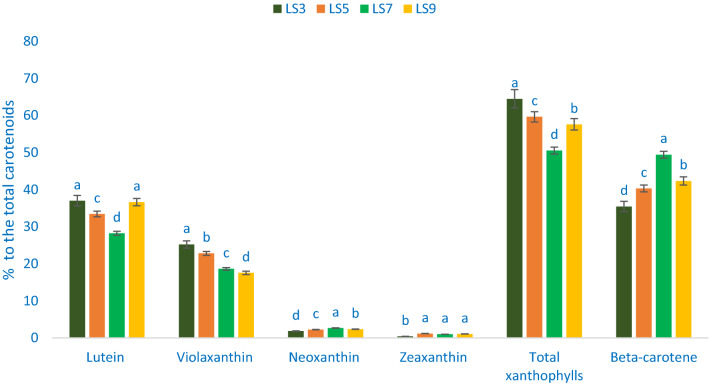
Percentage of zeaxanthin, lutein, violaxanthin, neoxanthin, total xanthophyll, and β-carotene to the total carotenoids in four selected *A. gangeticus* leafy vegetables, different letters in the bar are differed significantly by Duncan Multiple Range Test ((P < 0.01), (n = 3).

Table 2 shows the data on the λmax, molecular ion, retention time, main fragment ions in MS^2^, and identified carotenoid compounds. The values of carotenoid compounds from four accessions (LS3, LS5, LS7, and LS9) separated through LC were compared with standard carotenoid compound masses with respective peaks of the compounds. In *A. gangeticus* leaves, a total of five carotenoid compounds were identified. Across them, four were identified as xanthophylls such as neoxanthin, violaxanthin, zeaxanthin, and lutein) and one was identified as pro-vitamin A (β-carotene). Figures 3 and 4 show the identified carotenoid profiles including total xanthophylls and total carotenoids and % of xanthophylls (zeaxanthin neoxanthin, violaxanthin, and lutein), pro-vitamin A (β-carotene), total xanthophylls to total carotenoids of four selected *A. gangeticus* leaves, respectively.

**Table 2:** Retention time, wavelengths of maximum absorption in the visible region (λ_max_), mass spectral data and tentative identification of carotenoid profiles in four selected *A. gangeticus* leafy vegetables.

Peak no	Retention time (min)	λ_max_ (nm)	Molecular ion [M−H]^−^ (m/z)	MS^2^ (m/z)	Identity of tentative carotenoids
1	2.52	450	438.54	438.62	Neoxanthin
2	2.63	450	446.43	446.37	Violaxanthin
3	3.84	450	445.22	445.17	Lutein
4	4.28	450	452.63	452.59	Zeaxanthin
6	20.16	450	449.52	449.46	β-carotene

Across xanthophylls, the most prominent identified carotenoid was lutein, followed by violaxanthin, while the zeaxanthin and neoxanthin contents were very low in *A. gangeticus* accessions (Fig. 3). We noticed much higher Zeaxanthin, lutein, β-carotene, total xanthophylls, neoxanthin, and total carotenoid contents of *A. gangeticus* accession compared to the contents of vegetable amaranth accession of Raju et al.^57^. Zeaxanthin, lutein, β-carotene, total xanthophylls, neoxanthin, violaxanthin, and total carotenoid contents 0.36 to 1.34, 31.42 to 46.72, 31.42 to 46.72, 31.42 to 46.72, 1.58 to 3.62, 17.89 to 25.30, and 84.87 to 135.67 mg 100 g^−1^, respectively (Fig. 3). The highest total xanthophylls (73.36 mg 100 g^−1^) and lutein (46.72 mg 100 g^−1^) were recorded in the accession LS9, followed by the accession LS7. On the other hand, the accession LS5 exhibited the lowest total xanthophylls (46.81 mg 100 g^−1^) and lutein (26.34 mg 100 g^−1^). The accession LS7 showed the highest β-carotene (67.12 mg 100 g^−1^), violaxanthin (25.30 mg 100 g^−1^), and total carotenoids (135.67 mg 100 g^−1^) followed by the accession LS9. Conversely, the accession LS5 had the lowest total carotenoids (78.45 mg 100 g^−1^) and violaxanthin (17.89 mg 100 g^−1^), whereas the accession LS3 had the lowest β-carotene (30.11 mg 100 g^−1^) (Fig. 3). The accession LS7 exhibited the highest neoxanthin (3.62 mg 100 g^−1^) followed by the accession LS9. In contrast, the accessions LS3 and LS5 showed the lowest neoxanthin (1.58 and 1.75 mg 100 g^−1^). The highest zeaxanthin was recorded in the accession LS9 (1.34 mg 100 g^−1^) and LS7 (1.34 mg 100 g^−1^), while the accession LS3 had the lowest zeaxanthin (0.36 mg 100 g^−1^) (Fig. 3).

Percentage of lutein, violaxanthin, neoxanthin, zeaxanthin, total xanthophylls, β-carotene, to total carotenoids ranged from 28.24 to 37.02, 17.56 to 25.22, 1.85 to 2.67, 0.43 to 1.18, 50.55 to 64.52, and 35.48 to 49.35 84.87 to 135.67 mg 100 g^−1^, respectively (Fig. 4). The accession LS7 demonstrated the highest percentage of violaxanthin (25.22%), and total xanthophylls (64.52%) to total carotenoids, albeit the accession LS3 and LS9 showed the highest percentage of lutein (37.02 and 36.66%). In contrast, the lowest percentage of lutein (28.24%) and total xanthophylls (50.55%) was observed in the accession LS7, and the lowest percentage of violaxanthin (17.56%) was noticed in the accession LS9. Neoxanthin percentage was the highest in the accession LS7 (2.67%), followed by LS9, while the accession LS3 showed the lowest percentage of neoxanthin (1.85%). The accession LS5 exhibited the highest percentage of zeaxanthin (1.18%), which was statistically similar to the accession LS7 and LS9. Conversely, the lowest zeaxanthin was recorded in the accession LS3 (0.43%). The highest percentage of β-carotene was obtained from the accession LS7 (49.45%) followed by the accession LS9. In contrast, the accession LS3 showed the highest percentage of β-carotene (35.48) (Fig. 4).

The results of total carotenoids of our study corroborated the results of Khanam and Oba^37^^.^ They observed higher carotenoids in the red amaranth accession compared to green amaranth*.* The vegetable amaranth LS7 and LS9 contained higher lutein, violaxanthin, neoxanthin, zeaxanthin, total xanthophylls, β-carotene, and total carotenoids compared to the accession LS3 and LS5. Hence, the carotenoid profiles of vegetable amaranth accession could play a crucial role in the detoxification of ROS in the human body and considered as an essential parameter for consumers as it acts as an antiaging and many degenerative human diseases^17,21^. Our result showed that the vegetable amaranth accession is an excellent source of lutein, violaxanthin, neoxanthin, zeaxanthin, total xanthophylls, β-carotene, and total carotenoids among leafy vegetables that has important free radical-scavenging activity^19^.

In this study, we found considerable pigments profile such as betacyanins, betalains, betaxanthins, and carotenoid profiles such as lutein, violaxanthin, neoxanthin, zeaxanthin, total xanthophylls, β-carotene in *A. gangeticus* leafy vegetable accession. The results of total carotenoids of our study corroborated with the results of Khanam and Oba^58^ and Raju et al.^57^, where they observed higher carotenoids in the red amaranth accession compared to green amaranth and *A. gangeticus*, respectively*.* The accession LS7 and LS9 contained higher lutein, violaxanthin, neoxanthin, zeaxanthin, total xanthophylls, β-carotene, and total carotenoid compared to other accessions. Hence, the carotenoid profiles of vegetable amaranth accession could play a crucial role in the detoxification of ROS in the human body and considered as an essential parameter for consumers as it acts as an antiaging and many degenerative human diseases^17,21^. Our result showed that the *A. gangeticus* accession is an excellent source of lutein, violaxanthin, neoxanthin, zeaxanthin, total xanthophylls, β-carotene, and total carotenoids among leafy vegetables that has important free radical-scavenging activity^19^. *A. gangeticus* accessions LS7 and LS9 had high carotenoid profiles, such as zeaxanthin, lutein, violaxanthin, neoxanthin, total xanthophylls, β-carotene, and total carotenoids content. The genotypes LS7 and LS9 might be used as carotenoids enriched high-yielding varieties for drink purposes. The present investigation revealed that these two accessions have abundant carotenoids that offered new insight for detail pharmacological study.

### Betaxanthins, betalains, and radical quenching capacity

**Figure 5:**
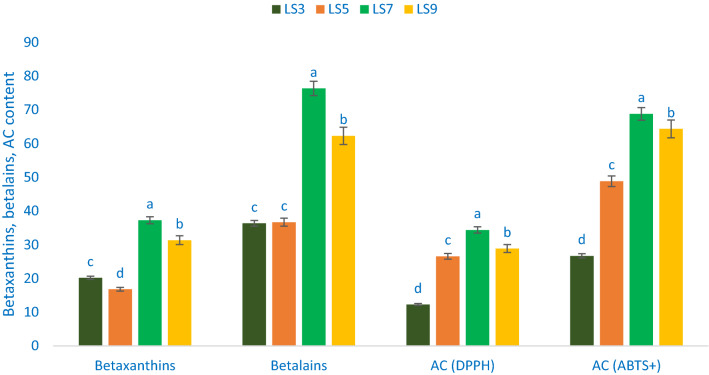
Betaxanthins, betalains, and antioxidant capacity in four selected *A. gangeticus* leafy vegetables, betaxanthins (mg 100 g^−1^ FW), betalains (mg 100 g^−1^ FW), AC (DPPH) = Antioxidant capacity (DPPH) (TEAC µg g^−1^ DW), AC (ABTS^+^) = Antioxidant capacity (ABTS^+^) (TEAC µg g^−1^ DW), different letters in the blue and green bars are differed significantly by Duncan Multiple Range Test ((P < 0.01), (n = 3).

Betalains, betaxanthins, and antioxidant capacity (AC) varied significantly among the studied *A. gangeticus* leafy vegetables accession (Fig. 5). Betaxanthins exhibited much pronounced variation in terms of accessions. Betaxanthins ranged from 16.78 mg 100 g^−1^ FW in the accession LS5 to 37.25 mg 100 g^−1^ FW in the accession LS7. Similarly, betalains ranged from 36.35 mg 100 g^−1^ FW in the accession LS3 to 76.36 mg 100 g^−1^ FW in the accession LS7. AC (DPPH) ranged from 12.27 TEAC µg g^−1^ DW (LS3) to 34.38 TEAC µg g^−1^ DW (LS7). The highest AC (DPPH) was recorded in the accession LS7 followed by LS9 and LS5. In contrast, the accession LS3 had the lowest AC (DPPH). AC (ABTS^+^) ranged from 26.69 TEAC µg g^−1^ DW to 68.79 TEAC µg g^−1^ DW. The *A. gangeticus* accession LS7 had the highest AC (ABTS^+^), followed by LS9. In contrast, AC (ABTS^+^) was the lowest in LS3. These findings were corroborative to the results of Khanam and Oba^58^, where they observed higher total betaxanthins, betalains content, and AC in the red amaranth accession compared to green amaranth*.* The *A. gangeticus* accession LS7 and LS9 contained higher betaxanthins, betalains, and AC than the accession LS3 and LS5. Hence, these antioxidant constituents of *A. gangeticus* accession played a crucial role in the detoxification of ROS in the human body and are considered an essential parameter for consumers. It acts as an antiaging and many degenerative human diseases^17,21^. The present findings revealed that the *A. gangeticus* accessions exhibited an excellent source of betalains, betaxanthins, and AC (DPPH & ABTS^+^) among leafy vegetables that have important free radical-scavenging activity^19^.

In this study, we found considerable color attributes, betacyanin profiles, carotenoid profiles, betalains, betaxanthins, and AC in the *A. gangeticus* accessions. The present findings were corroborated by the results of Khanam and Oba^58^, where they observed higher AC, betacyanins, betaxanthins, betalains, total carotenoids in the red amaranth accession compared to green amaranth*.* Betacyanin, total carotenoids, AC (ABTS^+^), and AC (DPPH) obtained in this study corroborated with the results of Khanam et al.^59^ in *A. tricolor.* We found two to three-fold greater β-carotene contents in red color accessions compared to the β-carotene contents of *A. gangeticus* accession of Raju et al.^57^. The leaf β-carotene contents of red color accessions two to three-fold and green color accession were 50% greater than the β-carotene contents of the leaves of *A. caudatus*^21^. Li et al.^60^ noticed the highest total AC (FRAP and ORAC methods) in *A. hypochondriacus* leaves compared to *A. caudatus* leaves. They also reported that leaves had the most increased AC (FRAP) than different parts of plants (seed, stalks, sprouts, flowers). It is difficult to compare our present results due to the difference in extraction and estimation methods and standard references. The accessions LS7 and LS9 had high color attributes, betacyanins, carotenoid profiles, betaxanthins, betalains, and AC. The antioxidant profile enriched high-yielding genotypes LS7 and LS9 can be used as drinks. The accessions LS7 and LS9 had high carotenoid profiles that could be used as high carotenoid profiles enriched varieties for drink purposes. The present investigation revealed that these accessions could offer enormous prospects for feeding the antioxidant-deficient community.

### Composition of proximate

**Figure 6:**
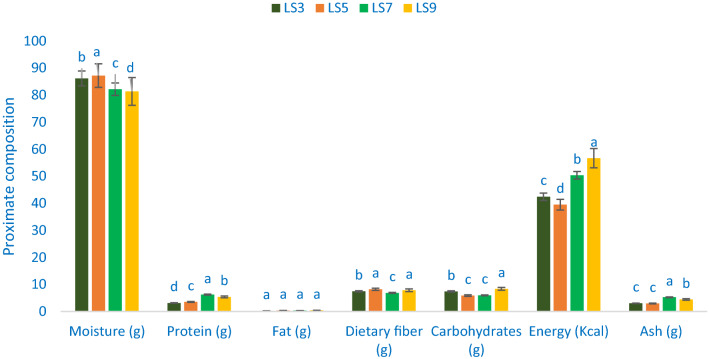
Proximate compositions (per 100 g^−1^ FW) in four selected *A. gangeticus* leafy vegetables; different letters are differed significantly by Duncan Multiple Range Test (P < 0.01), (n = 3).

The composition of proximate of *A. gangeticus* accessions is shown in Fig. 6. The range of moisture content of leaves was 81.35 g 100 g^−1^ to 87.24 g 100 g^−1^. LS5 showed the highest moisture content of 87.24 g 100 g^−1^), whereas LS9 exhibited the lowest moisture content (81.35 g 100 g^−1^ FW). As a higher dry matter of leaf confirm lower moisture contents, two genotypes (19–18% dry matter) had considerable dry biomass. The maturity is strongly associated with the leaf moisture content. The results obtained in this study were corroborated by the reports of *A. tricolor* and sweet potato leaves by Sarker and Oba^24^ and Sun et al.^61^, respectively. Significant and noticeable variations in protein content were observed for the accessions of *A. gangeticus*. The highest protein content was obtained from the genotype LS7 (6.24 g 100 g^−1^) followed by LS9. In contrast, the genotype LS3 had the lowest protein content (3.15 g 100 g^−1^). Vegetable amaranth is one of the vital sources of protein for poor people and vegetarians of developing countries. The protein content of *A. gangeticus* accessions was much higher than *A. tricolor* (1.26%) in our earlier study^2^. The selected *A. gangeticus* vegetable amaranths had no significant variations in fat content. The range of fat content was 0.23 to 0.41 g 100 g^−1^ FW. These results were corroborative to the results of *A. tricolor*^24^ and sweet potato^61^, respectively.

The highest carbohydrates were recorded in the genotype LS9 (8.39 g 100 g^−1^) followed by LS3. Conversely, the carbohydrate content was the lowest in LS5 (5.88 g 100 g^−1^) and LS7 (5.97 g 100 g^−1^). The highest energy was recorded in the genotype LS9 (56.68 kcal) followed by LS7. However, the lowest energy was obtained from the genotype LS5 (39.38 kcal). The highest ash content was noticed in LS7 (5.26 g 100 g^−1^) followed by LS9. On the other hand, the lowest ash content was noted in LS5 and LS3 (2.98 and 3.03 g 100 g^−1^). The least variations were noted for dietary fiber across four selected *A. gangeticus* accessions. The highest dietary fiber was obtained from the accessions LS5 and LS9 (8.22 and 7.85 g 100 g^−1^ FW) followed by LS3, whereas dietary fiber content was the lowest in LS7 (6.88 g 100 g^−1^ FW). Dietary fiber had a tremendous contribution to the cure of constipation, the increment of digestibility, and palatability^4^. The current results indicated that leaves of *A. gangeticus* accessions have abundant moisture, protein, dietary fiber, and carbohydrates. The present study is corroborative to the results of our earlier study^24^. The results of dietary fiber and carbohydrate were corroborative to our previous studies of red morph amaranth^14^, weedy amaranth^10^, green morph amaranth^13^, stem amaranth^11^, and *A. blitum*^12^. Whereas, dry matter contents of four amaranth accessions were greater than the dry matter contents of red morph amaranth^14^, weedy amaranth^10^, green morph amaranth^13^, stem amaranth^11^, and *A. blitum*^12^. The protein contents of these four amaranth accessions were greater than the protein contents of red morph amaranth^14^, green morph amaranth^13^, stem amaranth^11^, and *A. blitum*^12^.

### Minerals composition

**Figure 7:**
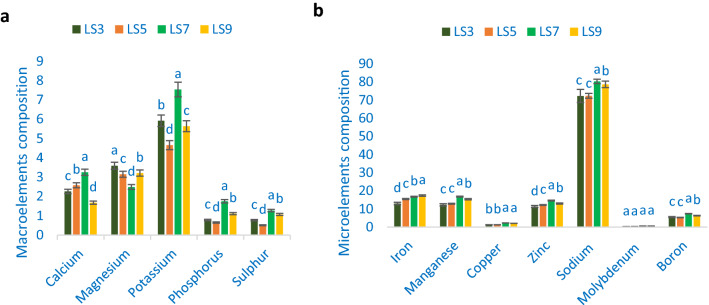
Minerals compositions (**a**) Macroelements (mg g^−1^ FW) (**b**) Microelements (µg g^−1^ FW) in four selected *A. gangeticus* leafy vegetables, different letters are differed significantly by Duncan Multiple Range Test (P < 0.01), (n = 3).

Minerals composition of *A. gangeticus* accessions is shown in Fig. 7. The potassium content ranged from 4.66 mg g^−1^ to 7.54 mg g^−1^. The highest potassium content was recorded in the genotypes LS7. Conversely, the lowest potassium content was observed in the genotype LS5. The range of calcium was 1.68 to 3.25 mg g^−1^. The highest calcium content was obtained from the genotype LS7, whereas the lowest calcium content was noted in the genotype LS9. LS3 had the highest magnesium content (3.59 mg g^−1^) followed by LS5 and LS9. On the other hand, the lowest magnesium was recorded in LS7 (2.49 mg g^−1^). The range of phosphorus and sulfur content of vegetable amaranth leaves was 0.65–1.75 and 0.51–1.27 mg g^−1^. The highest phosphorus and sulfur content The genotype LS7 exhibited, while the genotype LS5 showed the lowest phosphorus and sulfur content. Adequate calcium (3.25 mg g^−1^), potassium (7.54 mg g^−1^), sulfur (1.27 mg g^−1^), magnesium (3.59 mg g^−1^), and phosphorus (1.75 mg g^−1^) were observed in *A. gangeticus* accessions. Chakrabarty et al.^6^ in *A. lividus* and Sarker and Oba^24^ in *A. tricolor* also observed similar results in different amaranths. Jimenez-Aguiar and Grusak^62^ noted abundant potassium, calcium, magnesium, phosphorus, and sulfur. They also reported pronounced potassium, calcium, magnesium, phosphorus, and sulfur in amaranth compared to spinach, black nightshade, spider flower, and kale.

Adequate iron and manganese content was recorded in *A. gangeticus* accessions. The highest iron content was observed in the genotype LS9 (17.35 µg g^−1^), followed by LS7 and LS5. Conversely, the lowest iron content was recorded in the genotype LS3 (12.99 µg g^−1^). In this study, manganese ranged from 12.25 to 16.77 µg g^−1^. The highest manganese content was noted in the genotype LS7, whereas the lowest manganese content was obtained from the genotype LS3. Copper content had significant and notable variations across the *A. gangeticus* accessions (1.27–2.26 µg g^−1^). The highest copper content was observed in LS7, followed by LS9. In contrast, the lowest copper content was obtained from the genotype LS3 and LS5, respectively. Adequate zinc, sodium, and boron were recorded across the *A. gangeticus* accessions. The range of zinc, sodium, and boron content was 11.33–14.61, 72.24–80.28, and 5.27–7.36 µg g^−1^. The highest zinc, sodium, and boron were recorded in LS7, whereas the lowest zinc and sodium were obtained from LS3, and the lowest boron content was recorded in LS5. The range of molybdenum content was 0.26–0.57 µg g^−1^. The highest molybdenum content was observed in the genotypes LS7, whereas the lowest molybdenum content was noted in LS3. *A. gangeticus* accessions contained higher zinc and iron content than the cassava leaves^63^ and beach pea^64^. Adequate iron (17.35 µg g^−1^), copper (2.26 µg g^−1^), manganese (16.77 µg g^−1^), sodium (80.28 µg g^−1^), zinc (14.61 µg g^−1^), boron (7.36 µg g^−1^), and molybdenum (0.57 µg g^−1^) were recorded in *A. gangeticus* accessions. Earlier abundant iron, manganese, copper, zinc sodium, molybdenum, and boron were noted in different amaranths^62^. The leaves of amaranth had pronounced manganese, iron, zinc, and copper in than spinach, black nightshade, spider flower, and kale. The obtained potassium from these accessions was corroborative to previous studies of green morph amaranth^13^, whereas calcium recorded in these accessions was greater than red morph amaranth^14^, stem amaranth^11^, and *A. blitum*^12^. High phosphorus and sodium were observed compared to weedy amaranth^10^. Likewise, magnesium, zinc, and iron observed in the current study were much pronounced than red morph amaranth^14^, green morph amaranth^13^, stem amaranth^11^, and *A. blitum*^12^. High copper content was obtained from the present study, which is greater than the earlier study of green morph amaranth^13^, and manganese of the current study was greater than weedy amaranth^10^, green morph amaranth^13^. Hence, these selected advance lines could contribute as high minerals enriched genotypes compared to our previously tested amaranth genotypes.

### The correlation coefficient study

The correlation of betacyanins, betaxanthins, betalains, and AC of *A. gangeticus* leafy vegetables are shown in Table 3. Total betacyanins and betaxanthins, betalains had highly significant positive associations among themselves, with total carotenoids, AC (DPPH and ABTS^+^). It revealed that total betacyanins, betaxanthins, and betalains, exhibited strong AC. Total xanthophylls, β-carotene, and total carotenoids had significant positive interrelationships with β-carotene, total carotenoids, AC (DPPH and ABTS^+^) that signify that major carotenoids had strong AC. The results of the present study corroborative to the results of our earlier study of drought and salt-stressed *A. tricolor*^24^.

**Table 3:** The correlation coefficient for total betacyanin, major carotenoids components, antioxidant constituents, and antioxidant capacity in four selected *A. gangeticus* leafy vegetables.

	Betaxanthins (mg 100 g^−1^ FW)	Betalains (mg 100 g^−1^ FW)	Total xanthophyll (mg 100 g^−1^ FW)	β-Carotene (mg 100 g^−1^ FW)	Total carotenoids (mg 100 g^−1^ FW)	AC (DPPH) (TEAC µg g^−1^ DW)	AC (ABTS^+^) (TEAC µg g^−1^ DW)
Total betacyanin	0.88**	0.93**	0.32	0.43	0.52*	0.75**	0.79**
Betaxanthins		0.97**	0.23	0.46	0.61*	0.89**	0.82**
Betalains			0.41	0.42	0.59*	0.84**	0.89**
Total xanthophyll				0.88**	0.87**	0.97**	0.83**
β-Carotene					0.98**	0.81**	0.82**
Total carotenoids						0.86**	0.89**
AC (DPPH)							0.97**

*AC (DPPH)* antioxidant capacity (DPPH), *AC (ABTS*^*+*^*)* Antioxidant capacity (ABTS^+^).

*Significant at 5% level.

**Significant at 1% level, (n = 3).

In conclusion, we identified betacyanin profiles containing amaranthine, iso-amaranthine, betanin, iso-betanin, carotenoid profiles containing zeaxanthin, lutein, violaxanthin, neoxanthin, total xanthophylls, β-carotene, and total carotenoids, betaxanthins, betalains, and AC (DPPH and ABTS^+^) in the *A. gangeticus* accessions. *A. gangeticus* vegetable amaranth genotypes contained ample proximate, and nutraceuticals, such as protein, carbohydrates, moisture, dietary fiber, iron, manganese, copper, zinc, sodium, molybdenum, boron, potassium, calcium, magnesium, phosphorus, sulfur. The correlation study revealed that all pigments of *A. gangeticus* had high AC. The present investigation revealed that these accessions exhibited excellent sources of antioxidants components with ROS quenching capability that offered huge prospects for detail pharmacological study. The baseline data on color attributes, betacyanins, carotenoids, betaxanthins, betalains, and AC obtained in the present study could contribute to the scientific evaluation of pharmacologically active principles in *A. gangeticus*. *A. gangeticus* accessions LS7 and LS9 had abundant color attributes, betacyanins, and carotenoid profiles, betaxanthins, betalains, proximate, nutraceuticals, and antioxidant potentiality. These two accessions LS7 and LS9 could be recommended as preferable cultivars for consumption as sources of phytopigments, nutraceuticals, and antioxidants.

## Methods

### Experimental materials

We selected four high yields and antioxidant potential *A. gangeticus* accessions from few accessions. The seeds of four advance genotypes were collected from the Department of Genetics and Plant Breeding of Bangabandhu Shiekh Mujibur Rahman Agricultural University. It is the first report on color attributes, betacyanin, carotenoid profiles, bioactive components, and antioxidants potentials in *A. gangeticus*.

### Design and layout

The experiment was executed in three replicates following a completely randomized block design (RCBD) at Bangabandhu Sheikh Mujibur Rahman Agricultural University. Each genotype was grown in a 1 m^2^ experimental plot following 20 cm and 5 cm distance between rows and plants, respectively. The experimental site is located about 24°23′ N latitude 90°08′ E longitude, in the Agroecological Zones 28 (center of the Madhupur Tract), with an average elevation of 8.4 msl. The site is high land and falls under subtropical climatic conditions with mean winter temperatures of 18 °C and summer temperatures of 29 °C. The soil characteristics of the experimental field are silty clay with low in organic matter (0.87%), slightly acidic (pH 6.4), exchangeable K (0.13 cmol kg^−1^), and total N (0.09%). The soil Zn and P content are above the critical level, while S content is a critical level (Critical levels of Zn, S, and P are 0.2, 14, and 14 mg kg^−1^, respectively and the K level is 0.2 cmol kg^−1^).

### Intercultural practices

We applied the recommended compost and fertilizer doses. At the time of land preparation, 10 ton ha^−1^ compost was applied. Triple superphosphate, urea, gypsum, and murate of potash were applied at 100, 200, 30, and 150 kg ha^−1^, respectively. The exact plant spacing in a row was maintained by thinning the row properly. Weeds were regularly removed through proper weeding and hoeing. We provide regular irrigation in the experimental plots for retaining the appropriate growth of vegetable amaranth. We collected the leaf samples at 30 days old plant. Ten randomly selected plants were selected to harvest from each experimental unit. The leaves were immediately sampled from the harvested plants.

### Estimation of color attributes

We measured the color attributes C*, L*, b*, and a* using a color meter (TES-135A, Plus, Taiwan) in 15 replicates. The positive value of (+ b*) indicates yellowness, while the negative value of (− b*) indicates blueness. The positive value of (+ a*) suggests the degree of redness, while the negative value of (− a*) indicates greenness. L* indicates lightness, and the C* value indicates leaf color intensity designated as chroma. The chroma value was calculated using the formula, Chroma C* = (a^2^ + b^2^)^1/2^.

### Samples extraction for HPLC and LC–MS analysis

10 mL of 80% methanol containing 1% acetic acid was added in 1 g of leaves and homogenized thoroughly, and transferred to a 50 mL tightly capped test tube. The test tubes were placed in a shaker (Scientific Industries Inc., USA) for 15 h at 400 rpm. 0.45 µm filter (MILLEX-HV syringe filter, Millipore Corporation, Bedford, MA, USA) was used to filter the homogenized mixture. The mixture was centrifuged at 10,000 × g for 15 min. Betacyanin components were analyzed from the final filtrate. Betacyanin analysis in the samples could interfere through the precipitation of methanol with the proteins and other insoluble substances in the samples. Strata-X 33 µm Polymeric Reversed-Phase cartridges (Phenomenex, Torrance, CA, USA) were used to purify betacyanin. All extractions were done in triplicate independent samples.

### Betacyanin analysis through HPLC

The methods previously used in *A. spinosus*^54^ and *A. tricolor*^65^ were followed to determine betacyanin components in the *A. gangeticus* leaf sample using HPLC. The high-performance liquid chromatograph Shimadzu SCL10Avp, Kyoto, Japan, was equipped with a degasser (DGU-14A), an LC-10Avp binary pumps, and a detector (Shimadzu SPD-10Avp UV–Vis). A column (CTO-10AC (STR ODS-II, 150 × 4.6 mm I.D., Shinwa Chemical Industries, Ltd., Kyoto, Japan) was used to separate the betacyanin components. Pumping of binary mobile phase was performed with solvent B (acetonitrile) and solvent A (6% (v/v) acetic acid) in the water at the flow rate of 1 mL min^−1^ for 70 min. The system was run using a gradient program with solvent acetonitrile 0–15% for 45 min, 30–50% for 5 min, 15–30% for 15 min, and 50–100% for 5 min. The column temperature was maintained at 35 °C with an injection volume of 10 μL. The detector was set at 538 nm for the simultaneous monitoring of betacyanin. For identification of the compound, we compared retention time and UV–Vis spectra with their respective standards. We confirmed the betacyanin components through the mass spectrometry assay method. All samples were prepared and analyzed in triplicates. The results were expressed as mg 100 g^−1^ FW for betacyanin components. A JEOL AccuTOF (JMS-T100LP, JEOL Ltd., Tokyo, Japan) mass spectrometer fitted with a UV–Vis detector coupled online and an Agilent 1100 Series HPLC system with an ElectroSpray Ionization (ESI) source to analyze the mass spectrometry with negative ion mode with the column elutes in the range of m/z 0–1000 and needle voltage at − 2000 V. Extract constituents were identified by LC–MS-ESI analysis.

### Quantification of betacyanin components

Calibration curves of the respective standards were used to quantify individual betacyanin components. The betanin standard was dissolved in 80% methanol as stock solutions to 100 mg mL^−1^. Standard curves (10, 20, 40, 60, 80, and 100 mg mL^−1^) were prepared and used to quantify the individual betacyanin components. The retention times, UV spectral characteristics, and co-chromatography of samples spiked with commercially available standards were used to identify and match the betacyanin components. Betanin standard was used to prepare standard curves based on the equimolecular conversion for estimating amaranthine and iso-amaranthine in the different samples.

### Sample preparation for extraction of carotenoids

The fresh leaf samples were washed thoroughly, blotted dry, lyophilized to dryness. All precautions were taken to prevent any significant losses of carotenoids due to photo-oxidation and isomerization. Sampling was done with subdued lighting and temperature at 20 °C. The dry samples were ground through a mechanical blender. The powdered samples were kept in aluminum foil inside a self-sealing bag and stored below − 20 °C until further use. The samples were stored for one week.

### Extraction of carotenoids

Carotenoids were extracted according to the procedure described by Lakshminarayana et al.^66^. Carotenoids were extracted with ice-cold acetone until the samples became colorless. Rapid extraction in cold acetone was employed to reduce the possibility of carotenoid oxidation. The crude extract (50 mL) was taken in a separatory funnel; 100 mL of petroleum ether and 100 mL of aqueous sodium chloride (25%, w/v) were added; after mixing well, the upper layer was separated. The extraction was repeated three times (total volume: 250 mL). The extract was dried over anhydrous sodium sulfate (20 g) and filtered through Whatman No.1 filter paper. The filtrate was evaporated to dryness in a rotary evaporator at 35 °C and redissolved in a known volume of hexane. An aliquot (100 µL) of the extract was dried under a stream of nitrogen and the residue was redissolved in 1 mL of acetonitrile/methanol/dichloromethane (60:20: 20 v/v/v). Samples were analyzed by HPLC. Sample handling, homogenization, and extraction were carried out at 4 °C, under dim yellow light to minimize photo-isomerization and oxidation of carotenoids.

### HPLC analysis

The HPLC method previously described by Lakshminarayana et al.^66^ was followed to estimate carotenoid profiles in *A. gangeticus* leaf samples. A variable Shimadzu SPD-10Avp UV–Vis detector, LC-10Avp binary pumps, and a degasser (DGU-14A) were equipped with the HPLC system (Shimadzu SCL10Avp, Kyoto, Japan). Briefly, the carotenoids were separated on a CTO-10AC (STR ODS-II, 150 × 4.6 mm I.D., Shinwa Chemical Industries, Ltd., Kyoto, Japan) column. The carotenoids were separated using acetonitrile/methanol/dichloromethane (60:20:20, v/v/v) containing 0.1% ammonium acetate as a mobile phase. For HPLC analysis, 20 µL samples were injected under the isocratic condition at a flow rate of 1 mL min^−1^. The Shimadzu SPD-10Avp UV–Vis detector was set at 450 nm. We confirmed the peak of carotenoids by comparing their retention time of standard chromatograms recorded with a Shimadzu model LC-10Avp series equipped with SPD-10AVP detectors. At the same time, the characteristic spectrum record with a PDA detector was taken to confirm the λ_max_ values of these compounds. We quantified the carotenoid profiles estimating their peak areas to respective reference standards.

### Betaxanthins content measurement

The leaves of *A. gangeticus* leafy vegetables were extracted in 80% methyl alcohol, having 50 mM ascorbate to measure betaxanthins according to the method of Sarker and Oba^67^. The absorbance was taken at 475 nm using a spectrophotometer (Hitachi, Japan) to measure for betaxanthins. The results were expressed as the milligrams indicaxanthin equivalent per 100 g FW for betaxanthins.

### Radical quenching capacity assay

Thirty days old *A. gangeticus* leaves were harvested. For the antioxidant capacity assay, the samples were dried in a shady place. 1 g dried leaves were extracted with 40 mL of 90% aqueous methanol in a 100 mL tightly capped bottle. The extract was shaken in a water bath (Thomastant T-N22S, Thomas Kagaku Co. Ltd., Japan) for one h. Exactly 0.45 µm filter (MILLEX-HV syringe filter, Millipore Corporation, Bedford, MA, USA) was used to filter the homogenized mixture. The mixture was centrifuged at 10,000×*g* for 15 min. The filtered extract was used to determine antioxidant capacity.

**Figure 8:**
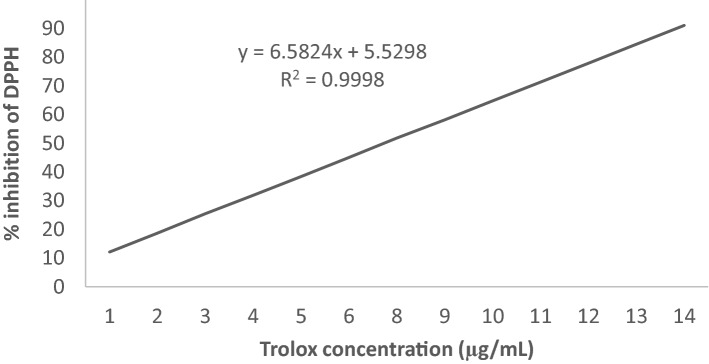
Calibration curve of the % inhibition of DPPH by Trolox versus the applied concentration range.

**Figure 9:**
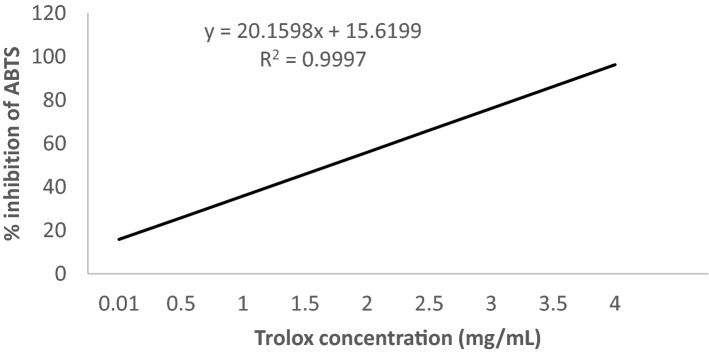
Calibration curve of the % inhibition of ABTS by Trolox versus the applied concentration range.

The antioxidant activity was estimated by the diphenyl-picrylhydrazyl (DPPH) radical degradation method^68^. In a test tube, 10 µL of diluted leaf extract was added to 1 mL of 250 µM DPPH solution and 4 mL of distilled water (in triplicate). In the dark place, the mixture was stood for 30 min. The absorbance was taken at 517 nm using a Hitachi spectrophotometer (Japan). ABTS^+^ assay was carried out using the method of Sarker et al.^69^. In the stock solutions, 2.6 mM potassium persulfate and 7.4 mM ABTS^+^ solution were used. The working solution was prepared by mixing two stock solutions equally. The mixture was allowed to react for 12 h at room temperature in the dark.. 150 μL sample of diluted leaf extract was added to 2850 μL of ABTS^+^ solution (1 mL ABTS^+^ solution mixed with 60 mL methanol) and allowed to react in the dark for two h. The absorbance was taken against methanol at 734 nm using a Hitachi spectrophotometer (Japan). The inhibition percentage of ABTS^+^ and DPPH corresponding to the control was utilized to measure the antioxidant activity following the equation:

$$ {\text{Antioxidant}}\;{\text{activity}}\;\left( \% \right) = \left( {{\text{A}}_{{\text{b}}} - {\text{A}}_{{\text{s}}} /{\text{A}}_{{\text{b}}} } \right) \times {1}00 $$

where A_b_ is the optical density of the control [150 μL methanol for AC (ABTS, 10 µL methanol for AC (DPPH)) instead of leaf extract] and A_s_ is the optical density of the test samples. The reference standard was Trolox. The equations Y = 6.5824X + 5.5298 with R^2^ = 0.9998 (Fig. 8) and Y = 20.1598X + 15.6199 with R^2^ = 0.9997 (Fig. 9) were obtained from Trolox standard calibration curve for DPPH and ABTS assay, respectively. Finally, the results were expressed as μg Trolox equivalent g^−1^ DW.

### Estimation of proximate composition

AOAC method was followed^70^ to estimate the ash, moisture, crude fat, fiber, crude protein contents, and gross energy. The nitrogen was calculated following the Micro-Kjeldahl method. Finally, measure crude protein was estimated by nitrogen × 6.25 (AOAC method 976.05). The ash, total moisture, crude protein, and crude fat (%) were subtracted from 100 for calculating carbohydrate (g 100 g^−1^ FW).

### Estimation of mineral composition

*A. gangeticus* accessions leaf samples were dried in an oven at 70 °C for 24 h. Dried samples were ground in a mill. We determined calcium, potassium, magnesium, phosphorus, sulfur, iron, manganese, copper, zinc, sodium, molybdenum, and boron from powdered leaves following the nitric-perchloric acid digestion method^71^. For this digestion, 400 mL HNO_3_ (65%), 10 mL H_2_SO_4_ (96%), and 40 mL HClO_4_ (70%) were poured into a 0.5 g dried leaf sample in the presence of carborundum beads. After digestion, P was measured by diluting the solution appropriately in triplicate following the ascorbic acid method. The antimony and ascorbic acid were added to the yellow-colored complex solution to convert it into a blue-colored phosphomolybdenum complex. Sarker and Oba^71^ method was followed to read the absorbance by atomic absorption spectrophotometry (AAS) (Hitachi, Japan) at 285.2 nm (magnesium), 76 6.5 nm (potassium), 880 nm (phosphorus), 258.056 nm (sulphur), 248.3 nm (iron), 279.5 nm (manganese), 422.7 nm (calcium), 213.9 nm (zinc), 589 nm (sodium), 324.8 nm (copper), 313.3 nm (molybdenum), and 430 nm (boron).

### Statistical analysis

The data analysis was performed using Statistix 8 software to obtain an analysis of variance (ANOVA)^72–74^. Duncan’s Multiple Range Test (DMRT) at a 1% level of probability was used to compare the means. The results were calculated as the mean ± SD of three separate replicates.

### Ethical statement

The lab and field experiments in this study were carried out as per guidelines and recommendations of “Biosafety Guidelines of Bangladesh” published by the Ministry of Environment and Forest, Government of the People’s Republic of Bangladesh (2005).

## Data Availability

All data generated or analysed during this study are included in this published article.
